# Navigated instrumentation and ligament tensioning device enhances initial gap acquisition during total knee arthroplasty procedure: A cadaveric study

**DOI:** 10.1002/jeo2.70107

**Published:** 2025-01-13

**Authors:** François Boux de Casson, Laurent Angibaud, Florian Kerveillant, Faustine Nogaret, Joris Ruffin, Léonard Duporté, Gérard Giordano, Louis Dagneaux

**Affiliations:** ^1^ Blue‐Ortho Meylan France; ^2^ Exactech Inc Gainesville Florida USA; ^3^ Department of Orthopaedic Surgery and Trauma University Center of Montpellier, University of Montpellier Montpellier France; ^4^ Joseph Ducuing Hospital Toulouse France; ^5^ Laboratoire de Mécanique et Génie Civil (LMGC) Montpellier University of Excellence Montpellier France

**Keywords:** alignment, experimental model, joint laxity, kinematics, ligament, tensor, TKA

## Abstract

**Purpose:**

Gap‐balanced total knee arthroplasty (TKA) technique relies on initial ligament evaluation, particularly in patient‐specific implantation using computer‐assisted technologies. This cadaveric study aimed to compare the reproducibility and reliability of medial and lateral gap measurements between manual stress testing and dynamic ligament balancer.

**Methods:**

Initial gap acquisitions were assessed from eight cadaveric knees (four specimens) during the same navigated TKA procedure by five differently skilled surgeons (three seniors and two juniors). Medial and lateral gaps were sequentially acquired from extension to maximum knee flexion, applying manual stress prior to any bone cuts (conventional technique), and using intra‐articular tensioning device placed between the tibial cut and the native femur (instrumented technique). Reproducibility was assessed using intraclass correlation coefficient (ICC), stratified by the measurement technique, the type of gaps and the operator experience. Differences in gaps (mm) between techniques were assessed using the Bland and Altmann method.

**Results:**

The instrumented technique showed higher ICCs than the conventional technique for medial and lateral gaps (0.87 vs. 0.60, *P* = 0.002, and 0.92 vs. 0.25, *p* < 0.0001, respectively), and showed no difference in ICCs between medial and lateral gap acquisitions (0.87 vs. 0.92, *p* = 0.8). Senior surgeons achieved higher ICCs than juniors, while non‐significant with both techniques. Differences in gaps between techniques increased with knee flexion angle (0.8, 2.8 and 3.5 mm at 10°, 45° and 90° of flexion angle, respectively) and decreased with the operator experience (*p* = 0.003).

**Conclusion:**

The instrumented balancing technique offered better reproducibility than using manual valgus and varus stress, when measuring medial and lateral gaps. Tensioning devices may play a significant role in enhancing initial gap acquisition, disregarding the flexion angle and the operator experience.

**Level of Evidence:**

Level IV (observational study involving cadaveric specimens).

**Abbreviations**
BA: Bland and Altman
CAOS: computer‐assisted orthopaedic surgery
ICC: intraclass correlation coefficient
TKA: total knee arthroplasty

## INTRODUCTION

Appropriate ligament balancing is one of the criteria for a successful total knee arthroplasty (TKA). The increasing use of unconstrained implants and the popularity of new alignment techniques have led to a heightened rate of revision for instability, which has been the second or third cause of TKA revision in several national registries for several years, including in the United States [1].

Achieving a proper soft‐tissue balance remains challenging in regard to the wide range of knee phenotypes and arthritic conditions [28]. As such, computer‐assisted orthopaedic surgery (CAOS) technologies have been developed to assist new alignment strategies focusing on the gap‐balancing technique. These technologies allow surgeons to evaluate knee gaps prior to planning the bone cuts, enabling adjustment of the bony cuts to achieve patient‐specific targets based on alignment and implant sizing considerations. Traditionally, medial and lateral gaps in extension and at 90° knee flexion are acquired by consecutively applying manual varus and valgus stress to the knee joint. However, this intraoperative planning depends on the reliability of the gap acquisitions [7, 14, 15], which may be questionable in this subjective technique [17]. Indeed, one surgeon may apply different magnitudes of varus and valgus forces and/or handling of the lower limb than another, leading to different gap assessments, and subsequently translating into different implant positioning, and tibial insert thickness selection for the same patient [10]. In addition, gap assessment during the entire arc of knee flexion, versus at a limited number of static positions, can be an asset for properly balancing the knee [12, 26].

To address this limitation, gap assessment can rely on a dynamic ligament balancer, distracting both knee compartments simultaneously while recording the gaps throughout the full arc of motion without applying manual varus or valgus stress. There are several types of devices, based on different mechanisms or actuations (e.g., mechanical or motorized) [3]. A particular class of balancer relates to intraarticular force‐controlled devices allowing simultaneous acquisition of both the medial and lateral gaps under distraction during the manipulation of the limb throughout the arc of motion in neutral alignment. As such, this experimental study aimed to compare gap measurements between the conventional (i.e., free‐handed technique with valgus and varus stress) and the instrumented balancing (i.e., using a dynamic balancer) techniques, with a particular emphasis on reproducibility and factors influencing the measurements' reliability. We hypothesized that the instrumented balancing technique would provide more reliable and reproducible gap measurements than the manual conventional technique, disregarding the operator's experience.

## MATERIALS AND METHODS

### Cadaver specimens

Eight lower limbs from four fresh frozen cadavers were studied. There were four women aged from 79 to 96 years at death (median = 92 ± 8 years). The lower limbs were harvested after approval by the local ethical committee. Each entire body was stored at −20°C, and then allowed to thaw at room temperature for 12 h before the experiments. Lower‐limb deformity included two knee varus, one knee valgus and one normoaxed. One specimen showed a slight contracture (~5°) on both legs. No cases of osteoarthritis of the knee were encountered.

### Data acquisition

**Figure 1:**
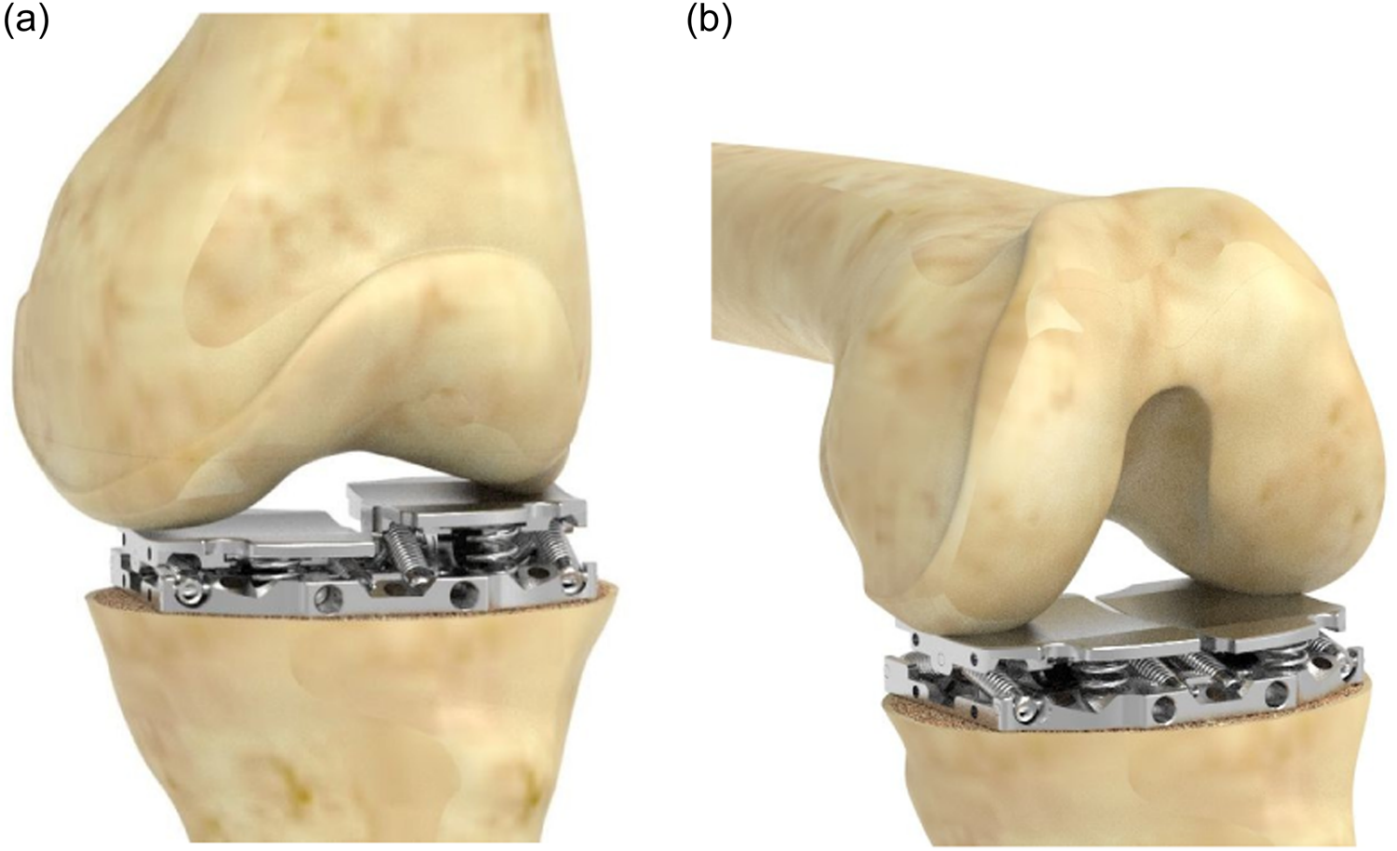
The dynamic ligament balancer was used after the proximal tibial cut, while recording the knee laxities throughout the arc of motion, from knee in extension (a) to knee in flexion (b).

All TKAs were performed by the same senior surgeon through a medial parapatellar approach using a CAOS system (ExactechGPS, Blue‐Ortho). After cruciate ligament resection and osteophytes removal, active femoral and tibial trackers were secured to each bone and anatomical landmarks were acquired to enable patient‐specific anatomic planification of a posterior stabilized implant (Optetrak Logic PS, Exactech). Medial and lateral gaps were sequentially acquired by the CAOS system from extension to maximum knee flexion, using the conventional technique prior to any bone cuts (Step 1). Then, the proximal tibial cut was performed perpendicular to the tibial mechanical axis, and the intra‐articular force‐controlled tensioning device (Newton, Exactech, see Figure 1) was placed between the tibial cut and the native femur. This mechanical tensioning device applies the same constant 90 N distraction force, independently on each compartment. After the release of the tensioning device, the lower limb was manipulated from extension to maximum flexion in neutral alignment (i.e., no varus or valgus stress) for the simultaneous acquisition of the medial and lateral gaps by the CAOS system, using the instrumented technique (Step 2). All acquisitions were done with the patella in place.

### Data processing

**Figure 2:**
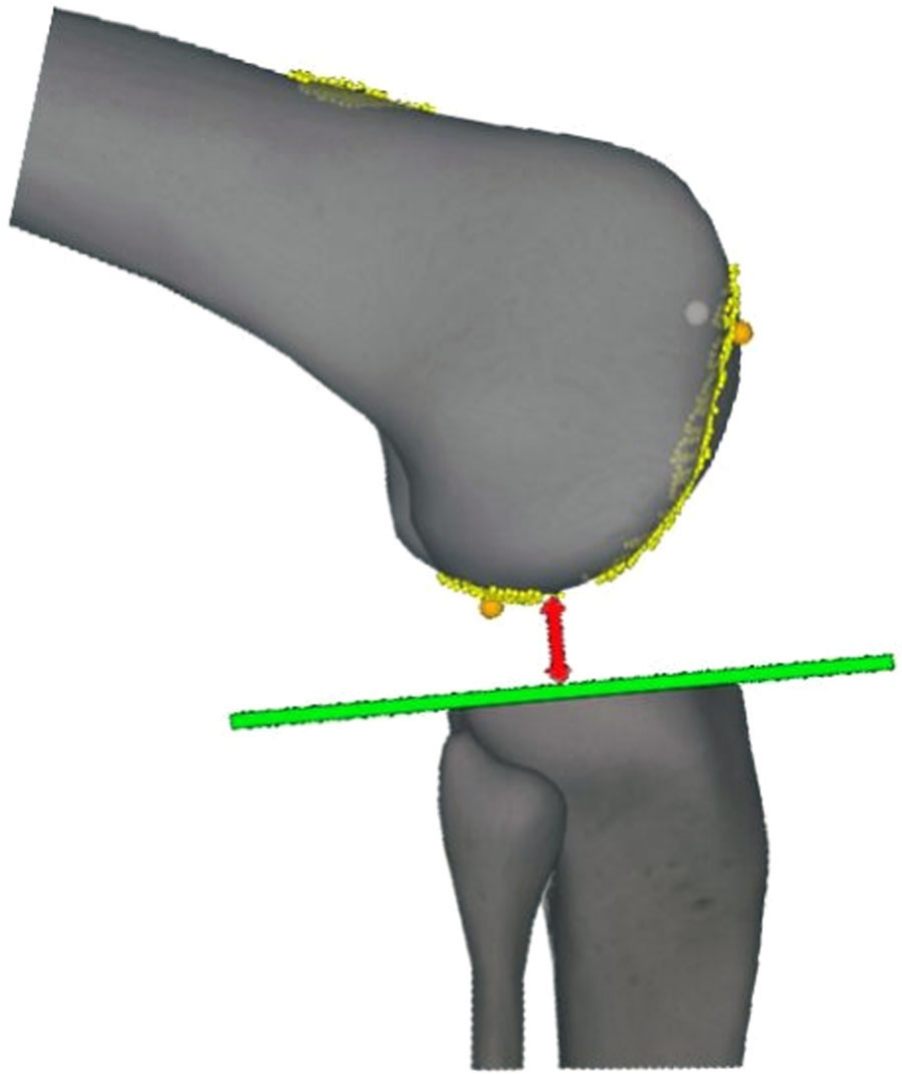
Gaps are computed by the navigation system as the smallest distance between the native femoral condyles surface and the plane of the tibia proximal cut.

Medial and lateral gaps were defined as the smallest distances between the medial and lateral condyles of the native femur and the checked tibial cut plane, respectively. For the conventional technique, distances were post‐processed after digitizing the tibial cut plane of the corresponding thickness (Figure 2). Gaps were recorded in the same coordinate system between acquisitions for each specimen, extracted continuously from 0° to 100° of knee flexion, and then plotted by each 5° flexion increments using house‐made software.

### Reproducibility analysis

Each gap was assessed with both techniques by five differently skilled operators, all right‐handed, including three senior surgeons (labelled S1, S2 and S3) and two junior surgeons (labelled J1 and J2). For each knee, each laxity measure (varus, valgus and instrumented) was repeated six times by each operator, alternating between operators, resulting in 960 gap acquisitions in total. Each evaluation was blinded from one operator to another. Intra‐operator and inter‐operator reproducibility were assessed by the intraclass correlation coefficient (ICC) with their 95% confidence intervals (95% CIs), stratified by the acquisition technique (conventional and instrumented), the type of gaps (both medial and lateral, lateral only and medial only), and the operator experience level (senior and junior). Inter‐operator reliability was assessed using a two‐way mixed effect mean of five raters with absolute agreement, while the intra‐operator reliability was assessed using a two‐way mixed effect, mean of six measurements with absolute agreement. The hypothesis was tested overall and specifically for medial and lateral gap measurements.

### Agreement analysis

To ensure whether measurement agreement between techniques was dependent on confounding factors (i.e., flexion angle, knee side, gap side and operator experience), Bland and Altman (BA) graphs [5] and their mean bias (expressed in millimetres) were assessed at 0°, 45° and 90° of knee flexion. The bias was defined as the gap from the instrumented technique minus the gap from the conventional technique. The impact of each confounding factor on bias between the two methods was measured using one‐way ANOVA (Tukey Post Hoc Test) for the knee flexion and using Student's or Welch's *t* tests (depending on whether variances were equivalent between groups) for the knee side, gap side and operator experience.

### Statistical analysis

All results were presented as means with ranges. Normal distribution was examined using the Shapiro–Wilk test, and the homogeneity of variance was examined using the *F* test. Differences in continuous variables were analyzed using the unpaired t‐test for normally distributed variables or Welch's two‐sample *t* test for non‐normally distributed variables. Superiority in continuous variables was analyzed using one‐sided *t* test. A power analysis was conducted to validate the sample size for comparing ICC between the two techniques using unpaired *t* test. An effect size of 0.1 was deemed relevant according to reference [16]. With an *α* level of 0.05 and a desired power of 80%, it was calculated that 143 measures per group would be required to detect a statistically significant difference. This sample size ensures sufficient power to minimize the risk of a Type II error. This recommended volume was far exceeded. Statistical analyses were performed using R software (version 4.3.1, R Foundation for Statistical Computing [18]). The *p* value was set as <0.05 for significance.

### Ethical aspects

Each author certifies that his institution has approved the reporting of these cases and that all investigations were conducted in accordance with ethical research principles.

## RESULTS

### Inter‐operator reproducibility

**Figure 3:**
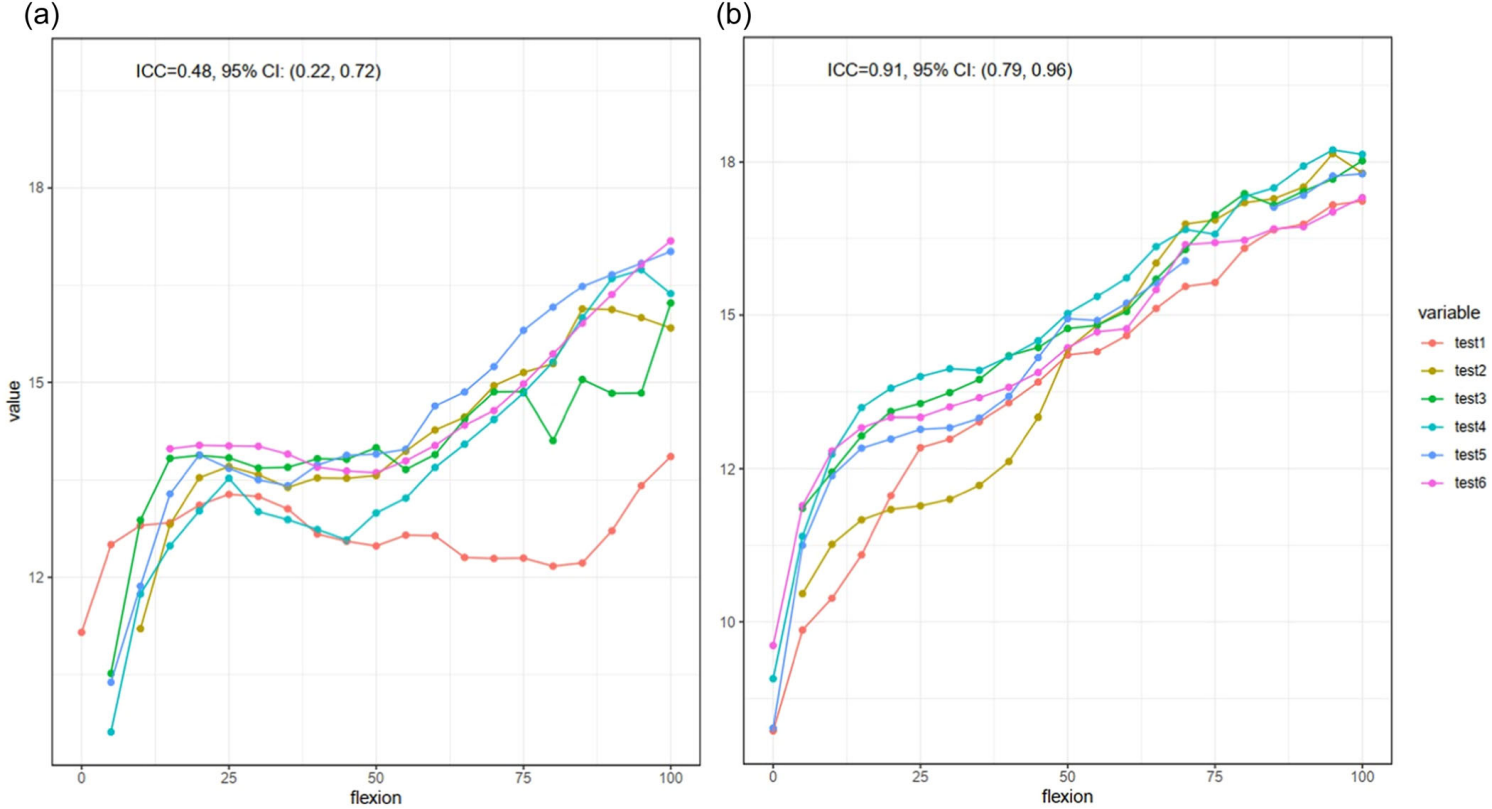
Mean lateral compartment gap measured along the leg flexion by each operator for specimen 4, left knee, by the conventional (a) and instrumented (b) techniques.

Overall, ICCs were significantly higher with the instrumented technique than with the conventional technique (0.90 vs. 0.43, *p* < 0.0001, respectively) (Figure 3 and Table 1). The same result was found when stratifying by medial gap (0.87 vs. 0.60, *p* = 0.002) and lateral gap (0.92 vs. 0.25, *p* < 0.0001). As such, a difference in reproducibility was found between medial and lateral gap acquisitions (0.60 vs. 0.25, *p* = 0.01) with the conventional technique, while no difference with the instrumented technique was found (0.87 vs. 0.92, *p* = 0.8) (Table 2). Senior surgeons achieved higher ICC than juniors, while non‐significant for either the conventional (0.55 vs. 0.39, *p* = 0.3, respectively) or the instrumented technique (0.92 vs. 0.90, *p* = 0.1, respectively) (Table 2).

**Table 1:** Inter‐operator and intra‐operator intraclass correlation coefficient (ICC) per technique of laxity measurements for all, varus and valgus tests.

	Mean ICC (95% CI)		One‐sided *t* test
	Conventional technique	Instrumented technique	
	Inter‐operator		
All tests	0.43 (0.23–0.60)	0.90 (0.76–0.96)	*p* < 0.0001
Varus tests (lat. gaps)	0.25 (0.09–0.43)	0.92 (0.81–0.97)	*p* < 0.0001
Valgus tests (med. gaps)	0.60 (0.37–0.76)	0.87 (0.71–0.95)	*p* = 0.002
	Intra‐operator		
All tests	0.51 (0.33–0.70)	0.78 (0.64–0.88)	*p* < 0.0001
Varus tests (lat. gaps)	0.40 (0.22–0.60)	0.84 (0.71–0.92)	*p* < 0.0001
Valgus tests (med. gaps)	0.63 (0.44–0.80)	0.71 (0.57–0.84)	*p* = 0.07

Abbreviation: CI, confidence interval.

**Table 2:** Inter‐operator and intra‐operator intraclass correlation coefficient (ICC) between measurement of lateral and medial gaps and between juniors and seniors surgeons.

	Mean ICC (95% CI)			Two‐sided *t* test
	Inter‐operator			
	Varus tests (lat. gaps)		Valgus tests (med. gaps)	
Conventional technique	0.25 (0.09–0.43)		0.60 (0.37–0.76)	*p* = 0.01
Instrumented technique	0.92 (0.81–0.97)		0.87 (0.71–0.95)	*p* = 0.8
	Juniors		Seniors	
Conventional technique	0.39 (0.08–0.62)		0.55 (0.27–0.72)	*p* = 0.3
Instrumented technique	0.90 (0.66–0.96)		0.92 (0.78–0.97)	*p* = 0.1
	Intra‐operator			
	Varus tests (lat. gaps)		Valgus tests (med. gaps)	
Conventional technique	0.40 (0.22–0.60)		0.63 (0.44–0.80)	*p* < 0.0001
Instrumented technique	0.84 (0.71–0.92)		0.71 (0.57–0.84)	*p* = 0.01
	Juniors		Seniors	
Conventional technique	0.53 (0.34–0.71)		0.50 (0.32–0.69)	*p* = 0.7
Instrumented technique	0.75 (0.61–0.87)		0.79 (0.66–0.89)	*p* = 0.2

### Intra‐operator reproducibility

**Figure 4:**
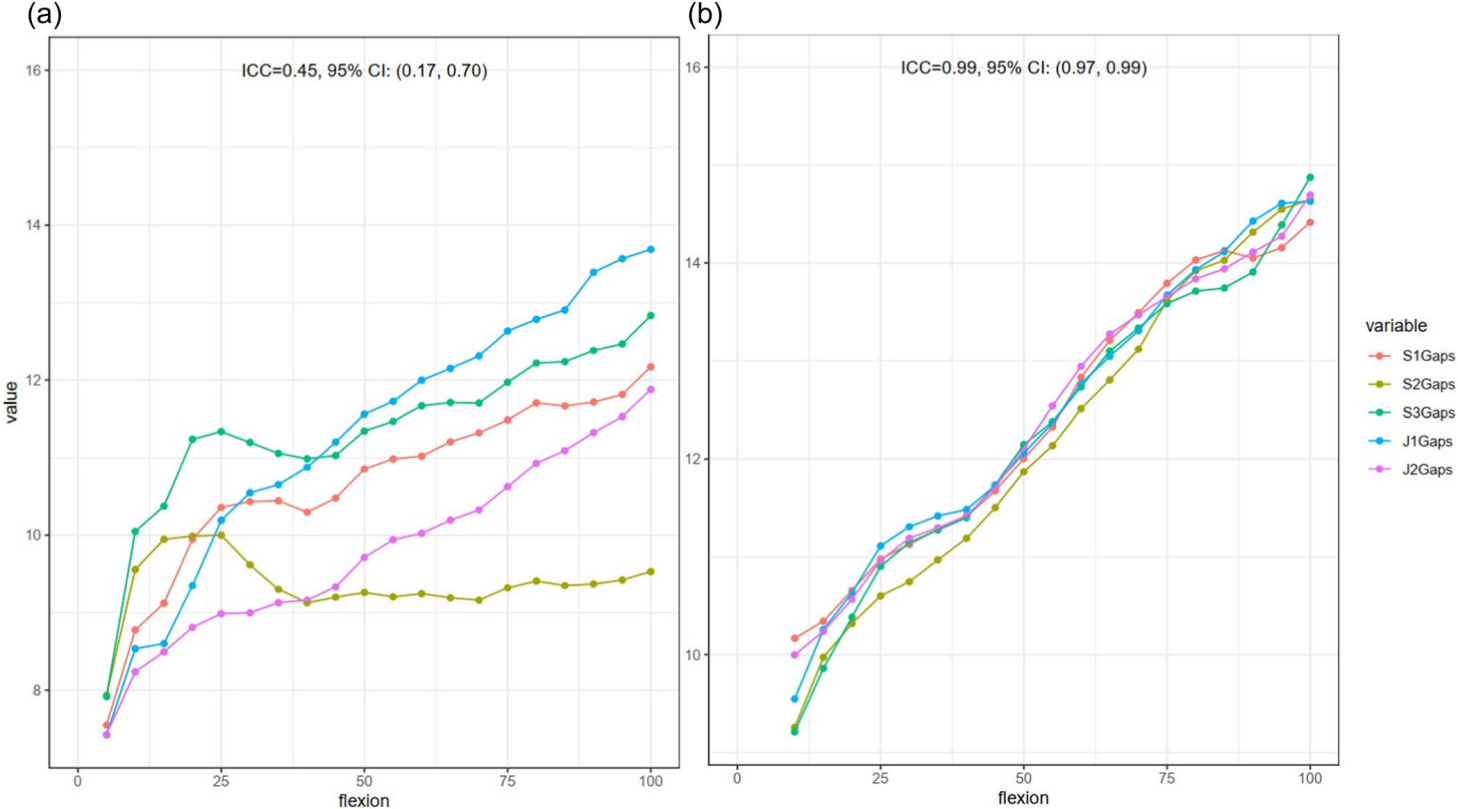
Repeated six acquisitions of the lateral compartment gap measured along the leg flexion by operator S3 for specimen 1, left knee, by the conventional (a) and instrumented (b) techniques.

Overall, ICCs were significantly higher with the instrumented technique than with the conventional technique (0.78 vs. 0.51, *p* < 0.0001) (Figure 4 and Table 1). The same result was found for lateral gap acquisition (0.84 vs. 0.63, *p* = 0.07). Medial gap acquisition was associated with higher ICC than lateral gap acquisition with the conventional technique, in contrast with the instrumented technique (0.71 vs. 0.84, *p* = 0.01, respectively) (Table 2). No significant differences in ICC were found regarding the operator experience for both techniques (conventional, *p* = 0.7 and instrumented, *p* = 0.2).

### Agreement results between techniques

Gaps from the instrumented technique were larger than those from the conventional technique at 10° (bias = 0.8 mm [−1.91 to 3.60]), 45° (bias = 2.1 mm [−0.44 to 4.69]) and 90° (bias = 3.5 mm [0.63–6.28]) (Table 3), and this bias increases significantly with the knee flexion angle (Table 4). Differences in gap measurements were found to be dependent on the knee side, showing significantly higher bias for right knees than left knees (1.7 ± 1.0 mm vs. 2.5 ± 1.7 mm, *p* = 0.049, respectively). Differences were dependent on operator experience, showing significantly higher bias for juniors than senior surgeons (2.7 ± 1.4 mm vs. 1.8 ± 1.5 mm; *p* = 0.003, respectively). Agreement between techniques was not influenced by the type of deformity (*p* > 0.77) or the gap side, showing similar bias between medial and lateral gaps (2.2 ± 1.6 mm vs. 2.1 ± 1.3 mm; *p* = 0.8, respectively) (Table 5).

**Table 3:** Bland and Altman bias and lower and upper limits of agreement (LOA) for gaps measurements at different flexion angles (instrumented technique minus conventional).

Flexion (°)	Bias mean (mm)	Lower LOA (mm)	Upper LOA (mm)
10	0.84 ± 0.67	−1.91 ± 1.67	3.60 ± 1.48
45	2.13 ± 0.81	−0.44 ± 1.57	4.69 ± 1.60
90	3.46 ± 1.33	0.63 ± 2.08	6.28 ± 1.42

**Table 4:** Bland and Altman bias for gaps measurements at different flexion angles between the two different techniques (instrumented minus conventional).

Flexion angle (°)			45	90
10	Mean difference of bias (mm)		−1.28	−2.62
			*p* = 0.002	*p* < 0.0001
45	Mean difference of bias (mm)		‐	−1.33
			‐	*p* = 0.001

*Note*: One‐way ANOVA—Tukey post hoc test.

**Table 5:** Bland and Altman bias (instrumented minus conventional) for gaps measurements comparing left and right knees, lateral and medial gaps and juniors and seniors surgeons.

Bland and Altman bias		
Bias mean (mm)		Two‐sided *t* test
Left knees	Right knees	
1.73 ± 0.98	2.55 ± 1.71	*p* = 0.049
Juniors	Seniors	
2.72 ± 1.45	1.80 ± 1.52	*p* = 0.003
Lateral gaps	Medial gaps	
2.19 ± 1.59	2.10 ± 1.31	*p* = 0.8

## DISCUSSION

This study investigated the reproducibility of two techniques measuring knee gaps throughout the full range of motion, during navigated tibia‐first TKA. The most important finding of this study was that the instrumented balancing technique provided a more reliable and reproducible gap measurement, along the full arc of flexion, than the manual conventional technique.

Disregarding the differences between alignment and implant philosophies [9], recent literature has supported that postoperative knee gaps can be predicted and achieved when using a CAOS system coupled with a dynamic ligament balancer [22]. As such, an accurate and reproducible laxity assessment remains essential to provide valuable quantitative information to perform patient‐specific surgical planning.

This study established that the instrumented balancing technique offered better reproducibility than using manual valgus/varus stress when measuring medial/lateral gaps. Considering the level of reliability [16], inter‐operator reproducibility ranged from poor with the conventional technique to excellent with the balancing technique, as expected. These results were consistent with those from previous work [2], highlighting the high degree of variability when assessing gaps from manual, inconsistent valgus/varus stress from operators. The instrumented device used in this study was designed for applying a distraction force of 90 N, independently to the medial and lateral compartments, which explains the improved reproducibility between operators. Several in‐vitro [19, 20, 23, 27] or in‐vivo [23, 24, 25] studies have analyzed the average forces required to tension the collateral ligaments of the knee. According to these studies, these forces vary between 50 and 130 N for the medial compartment and 50–100 N for the lateral compartment, with respective averages of 89 and 78 N. 90 N therefore represents an average force enabling measurement of the distances between the femoral and tibial spaces in extension and flexion without excessive or insufficient ligament tension.

The same trend was observed when focusing on lateral gap reproducibility, ranging from moderate with the conventional technique to good with the balancing technique. However, this trend was not observed for the medial gap reproducibility. These results also emphasize that the distracting device has played a significant role in enhancing initial lateral gap acquisition, which was less reproducible than the medial gap acquisition using conventional technique. Yet, accurate lateral gap assessment is crucial, as high lateral laxity in extension is associated with lower improvement in functional ability [13]. The distracting device can mitigate the factors influencing the lateral gap reproducibility, namely right or left‐handed surgeons and operator's experience.

Another factor influencing reproducibility in this study was the knee flexion. It is commonly accepted by surgeons that valgus or varus stress are challenging to manually apply consistently as the knee is moved from extension to deep flexion, and this trend was demonstrated experimentally by a recent cadaveric study [10]. As such, the difference in gap measures between techniques increased with knee flexion. As the dynamic ligament balancer aimed to apply the same distracting force to each compartment, disregarding the knee flexion angle, the BA bias indirectly captured the heterogenous varus/valgus forces applied with the conventional technique when flexing the knee. The dynamic ligament balancer also enhanced the gap acquisition when manipulating a right knee, as all the operators in this study were right‐handed and traditionally expressed more difficulties when applying varus stress on a right knee.

Concerning the influence of the operator's experience, BA analysis showed significantly greater differences between techniques for juniors, probably highlighting their difficulty to effectively manually distract the joints, notably in flexion. The instrumented technique eliminates the differences between juniors and seniors, with excellent inter‐operator ICC reproducibility for both groups.

Previous studies have shown near‐identical flexion gap measurement between surgeon vs. ligament tensor coupled with robot [29], or no difference in range of motion, reoperation rate or functional outcomes when compared to manually balanced TKA [21], but they focused only on flexion and extension gap assessments. Although it is beyond the scope of this study to assess the influence of a more precise and reproducible measurement of joint laxity on functional results, and studies have shown us to be cautious on this point [4], we do believe that assessing these gaps continuously over the entire arc of flexion can provide the practitioner with information that should make it possible to reduce mid‐flexion instabilities [11].

There are some limitations to discuss. First, cadaveric conditions cannot reflect the mechanical properties of ligamentous laxities in live subjects. Similarly, the number of manipulations carried out on the same specimen may have influenced the extent of deviation due to soft tissue stretching. This seems to be corroborated by the fact that intra‐operator reproducibility results were not as good as those for inter‐operator reproducibility, and probably increased the BA bias for the latest acquisitions. Additionally, this study was carried out on a relatively small number of cadaveric specimens with a low body mass index compared to standard TKA patients [6]. Third, the results found in this experimental study were linked to the tensioning device and the navigated instrumentation utilized in this study and cannot be extrapolated to all CAOS technologies or tensioning devices, even though some recent work has shown similar results [8].

## CONCLUSION

This study confirmed the hypothesis that the instrumented balancing technique would provide more reliable and reproducible medial and lateral gap measurements than using manual valgus and varus stress, disregarding the operator's experience. Tensioning devices may play a significant role in enhancing initial gap acquisition along the full arc of motion, regardless of the operator's experience.

## AUTHOR CONTRIBUTIONS

**François Boux de Casson, Laurent Angibaud, Florian Kerveillant**: Conceptualization; review analysis; writing—original draft. **Louis Dagneaux and Gérard Giordano**: Conceptualization; supervision; writing—review and editing. **Joris Ruffin, Faustine Nogaret and Léonard Duporté**: Experimental data; writing—review and editing.

## CONFLICT OF INTEREST STATEMENT

François Boux de Casson and Florian Kerveillant are employees of Blue‐Ortho, a subsidiary of Exactech. Laurent Angibaud is an employee of Exactech. Laurent Angibaud also serves an unpaid position as the Chair of the Scientific Committee for the Personalized Arthroplasty Society Gérard Giordano received consulting fees from Exactech during the conduct of the study. Louis Dagneaux reports a MUSE Explorer Grant from the University of Montpelier, a Research Fellowship Grant from SOFCOT, consulting fees from Zimmer Biomet, DePuy Synthes and Newclip Technics, speaker payments from Stryker and expenses from Zimmer Biomet, all of which are unrelated to this study. Louis Dagneaux also holds an unpaid position on the Board of Directors for SOFCOT, is on the Board of Directors for CAOS France, is the Director of the AFCP Scientific Council, and is Associate Editor of OTSR. Léonard Duporté, Joris Ruffin and Faustine Nogaret declare no conflict of interest.

## ETHICS STATEMENT

Local IRB from the University of Montpellier.

## Data Availability

The data that support the findings of this study are available from the corresponding author upon reasonable request.
